# Age and Sex-Related Changes in Retinal Function in the Vervet Monkey

**DOI:** 10.3390/cells11172751

**Published:** 2022-09-03

**Authors:** Catarina Micaelo-Fernandes, Joseph Bouskila, Roberta M. Palmour, Jean-François Bouchard, Maurice Ptito

**Affiliations:** 1 École d’Optométrie, Université de Montréal, Montreal, QC H3T 1P1, Canada; 2 Department of Psychiatry, McGill University, Montreal, QC H3A 1A1, Canada; 3 Behavioural Science Foundation, Basseterre KN 0101, Saint Kitts and Nevis; 4 Department of Neuroscience, University of Copenhagen, 2200 Copenhagen, Denmark

**Keywords:** electroretinogram, retina, cones, vervet monkey, age, aging, sex

## Abstract

Among the deficits in visual processing that accompany healthy aging, the earliest originate in the retina. Moreover, sex-related differences in retinal function have been increasingly recognized. To better understand the dynamics of the retinal aging trajectory, we used the light-adapted flicker electroretinogram (ERG) to functionally assess the state of the neuroretina in a large cohort of age- and sex-matched vervet monkeys (*N* = 35), aged 9 to 28 years old, with no signs of obvious ocular pathology. We primarily isolated the cone–bipolar axis by stimulating the retina with a standard intensity light flash (2.57 cd/s/m^2^) at eight different frequencies, ranging from 5 to 40 Hz. Sex-specific changes in the voltage and temporal characteristics of the flicker waveform were found in older individuals (21–28 years-old, *N* = 16), when compared to younger monkeys (9–20 years-old, *N* = 19), across all stimulus frequencies tested. Specifically, significantly prolonged implicit times were observed in older monkeys (*p* < 0.05), but a significant reduction of the amplitude of the response was only found in old male monkeys (*p* < 0.05). These changes might reflect ongoing degenerative processes targeting the retinal circuitry and the cone subsystem in particular. Altogether, our findings corroborate the existing literature in humans and other species, where aging detrimentally affects photopic retinal responses, and draw attention to the potential contribution of different hormonal environments.

## 1. Introduction

Throughout life, the retina undergoes degenerative structural changes that culminate in altered functional properties [1,2,3,4,5,6,7,8,9,10,11]. The electroretinogram is a validated, objective, reliable and reproducible method for assessing the global function of the neural retina. Moreover, it offers great flexibility in that it allows the manipulation of the light stimulus characteristics and the light adaptation state of the retina to isolate the electric activity of distinct cellular components.

On the whole, the bulk of studies that appraised the effects of healthy aging on retinal function in humans has observed a reduced amplitude and an augmented latency of the retinal response in older individuals, both in photopic and scotopic conditions [4,5,6,7,8,9,10,11]. Additionally, for decades, multiple studies have witnessed sex-related differences in retinal activity [4,5,8,12]. One of the first reports was published by Zeidler in 1959 [5]. This author observed a reduction in the b-wave potential for both sexes with aging. Interestingly, the average measured b-wave amplitude was slightly higher in women, and this difference was more pronounced in the third and fourth decades of life, after which the effect of aging appeared to be comparable between sexes. In a study involving 269 normal subjects, Birch and Anderson (1992) [8] found that the absolute amplitude values for women were superior to those of their age-matched men counterparts, although the observed age-associated decline in amplitude followed similar trends for both sexes.

The growing use of animal models in vision research urges for the controlled study of healthy experimental animals over their lifespan, to disentangle the functional decline occasioned by aging processes alone and to create a baseline for research aimed to evaluate the impact of age-related pathologies and treatments. In rodents there is also a well-corroborated loss of rod- and cone-mediated responses with age [13] that depends on sex, with function being more spared in females [14,15]. For non-human primates, however, the data are much scarcer.

Owing to their remarkable anatomical and physiological resemblance to humans, foveate diurnal non-human primates are an exceedingly pertinent model for eye research. In this regard, our team has a vast practice in experimenting with the vervet monkey. Among the many studies carried out over the last two decades, we have validated standard full-field photopic and scotopic ERG protocols for this species [16]. However, the sample of 15 monkeys used to obtain the normative values published therein was composed solely of young subjects (3 to 4 years old) and mostly females (13 out of 15). By recognizing the study of age- and sex-dependent effects/interactions as crucial to establish normative data, we performed full-field flicker ERG in a large cohort of healthy aging vervet monkeys to evaluate the cone–bipolar axis.

## 2. Materials and Methods

### 2.1. Subjects

A total of 36 vervet monkeys (*Chlorocebus sabaeus*) aged 9 to 28 years old with no previous records nor current obvious signs of ocular pathology were included in this study. One animal failed to achieve proper mydriasis which was later evident on its poor ERG traces and it was therefore excluded from all analyses. The remaining 35 animals were assigned to two different age groups: a group of younger animals, aged 9 to 20 years old (*N* = 19, 14.6 ± 3.9 years old, mean ± SD) and a group of older animals, 21 years old and above (*N* = 16, 22.9 ± 2.1 years old, mean ± SD). The animals were raised in the Behavioural Science Foundation (BSF, St. Kitts, West Indies) in naturalistic outdoor social settings. Animals in the BSF live in an enriched environment, with varied equipment to stimulate natural behaviours at their disposal (e.g., swings, perches, ladders, foraging and hiding places). They are fed with high-protein primate chow (Harlan Teklad, Madison, WI, USA), supplemented with fresh local produce and water is available ad libitum. The facility guarantees optimal Veterinary Care.

### 2.2. Animal Preparation

Monkeys were sedated with ketamine (10 mg/kg) (Troy Laboratories, Glendenning, New South Wales, Australia) and xylazine (1 mg/kg) (Lloyd Laboratories, Shenandoah, IA, USA) injected intramuscularly. Pupil dilation was achieved by the combined topical administration of 1% tropicamide and 2.5% phenylephrine hydrochloride (Alcon Laboratories, Fort Worth, TX, USA). Before placing the recording lens, proparacaine hydrochloride (0.5%) (Alcon Laboratories, Fort Worth, TX, USA) and methylcellulose (2.5%) (Akorn, Inc., Buffalo Grove, IL, USA) eye drops were applied to assure local anesthesia and corneal surface hydration, respectively.

### 2.3. Intraocular Pressure (IOP) Measurement

The TonoVet hand-held rebound tonometer (iCARE, Vantaa, Finland) was used to collect IOP measurements binocularly on mydriatic eyes, with the animals sedated and held in a sitting position. The upper eyelids were gently manually lifted during the readings.

### 2.4. Photopic Flicker Electroretinogram (ERG)

The ERG recordings were conducted using the UTAS E-3000 electrophysiology system (LKC Technologies, Gaithersburg, MD, USA), following the general recommendations of the ISCEV [17] and our previous standardized protocols in the vervet monkey [16,18]. The animal was kept unrestricted, laying on a tray in a prone position. For each eye, an ERG-Jet contact lens electrode (Diagnosys LLC, Lowell, MA, USA) coated with 1% carboxymethylcellulose sodium gel (Refresh Celluvisc, Allergan Inc., Markham, ON, Canada) was placed in alignment with the center of the cornea, in a way that the four built-in bumps on the external surface of the corneal electrodes helped keep the eyelids opened. Two gold disk reference electrodes were positioned bilaterally on the skin covering the temples and one ground skin electrode was attached to the middle of the forehead with Ten20 conductive EEG paste (Kappa Medical, Prescott, AZ, USA). After, the animal was placed facing the interior of a Ganzfeld with a steady luminance white background (30 cd/m^2^) for light adaptation. Stimuli consisted of standard intensity (2.57 cd/s/m^2^) multiple white flashes flickering at eight different frequencies (ranging from 5 to 40 Hz) delivered in full-field photopic conditions. Responses to different stimuli were recorded simultaneously for both eyes over six continuous 256 ms sweeps, amplified and bandpass-filtered (0.3 and 500 Hz cutoff frequencies). In total, each ERG recording session lasted about 15 min, comprising the time needed to set up the animals for the experiment. After, the animals were allowed to recover in individual cages, before being placed back in their prior group settings.

### 2.5. Statistical Analysis

A two-way mixed analysis of variance (ANOVA), with eye as within-subject factors and age group as between-subject factor, was conducted to compare the average intraocular pressure of the two age groups. To provide more detail into this comparison, a second analysis featuring sex as an additional between-subject factor was run.

Quantitative features of the flicker waveforms were automatically extracted by the UTAS E-3000 system associated EMWIN software. The data used for statistical comparisons was further refined by visual inspection of all the flicker waveforms for each individual recording sweep. Sweeps were discarded if any sign of technical flaws that could interfere with the calculation of the analyzed parameters was clearly identifiable (for example, the misplacement of the arrow marking the onset of a flash, marked irregularities of the baseline tracing). After this quality assessment, the amplitudes (measured from through to peak) and implicit times (measured from the flash onset to peak) of the selected waveforms were determined and averaged. The experimenter was blind to the characteristics (age and sex) of the subjects while carrying out this process. To examine the effect of aging on the parameters of the stimulus-response function, a three-way mixed ANOVA was performed, with flickering rate and eye as within-subject factors and age group as a between-subject factor. To investigate the possible interaction effect between aging and sex, sex was added as a between-subject factor on a subsequent analysis. Significant effects were scrutinized by conducting post hoc multiple comparisons with the Bonferroni correction when appropriate.

## 3. Results

### 3.1. Intraocular Pressure

**Figure 1:**
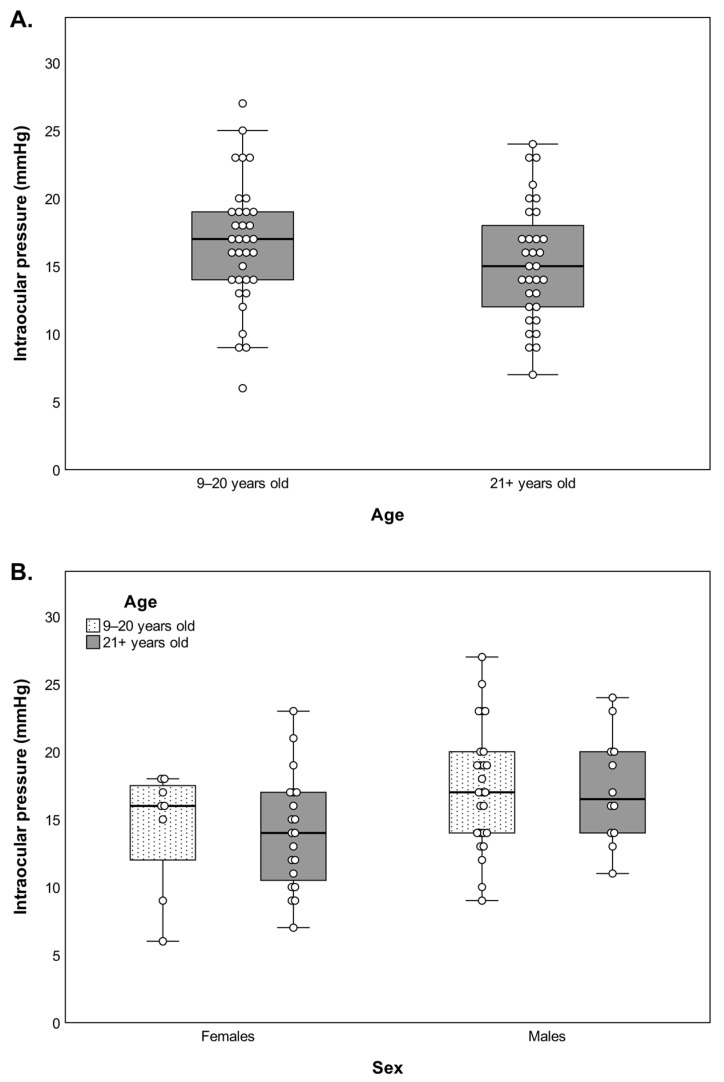
Distribution of the intraocular pressure in the tested monkeys by age (**A**) and by age and sex (**B**). No significant differences are found for any of the comparisons.

Intraocular pressure data were collected on 33 out of 35 monkeys before the ERG session. Overall, the distribution of the IOP values of both age groups resembled the one that is found in the healthy human population, averaging around 15 mmHg, with no significant differences between younger (16.6 ± 1.1 mmHg, mean ± SE) and older (15.3 ± 1.1 mmHg, mean ± SE) subjects (*F*(1,31) = 0.856, *p* = 0.362) (Figure 1A). Likewise, no significant main effect of sex was detected when this variable was incorporated in the analysis (*F*(1,29) = 3.595, *p* = 0.068) (Figure 1B).

### 3.2. Photopic Flicker ERG

**Figure 2:**
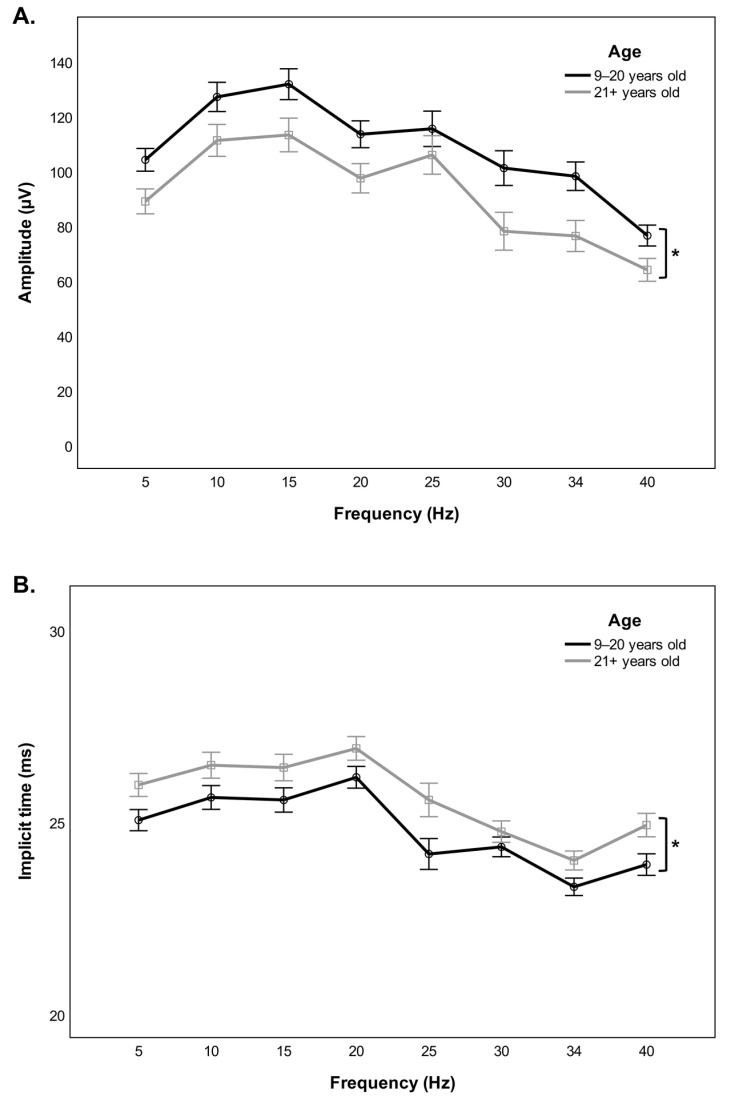
Frequency-amplitude (**A**) and frequency-implicit time (**B**) functions show differences in the flicker ERG stimulus-response characteristics between younger (9–20 years old) and older (21+ years old) monkeys. Responses were elicited at eight different flash frequencies. Error bars represent standard errors of the mean (SEM). *p < 0.05.

**Figure 3:**
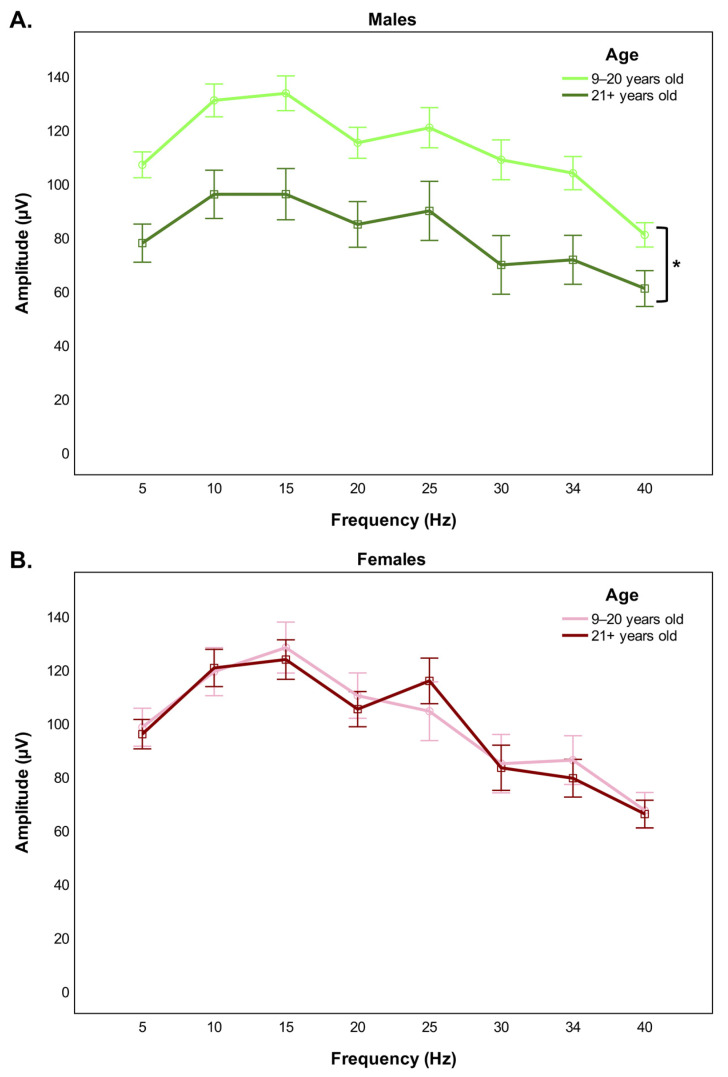
Sex-specific differences in the amplitude of the flicker ERG response with aging. The amplitude of the retinal response elicited at eight different flash frequencies is reduced in old males compared to young males (**A**), but it remains unaltered in old females compared to young females (**B**). Error bars represent standard errors of the mean (SEM). *p < 0.05.

**Figure 4:**
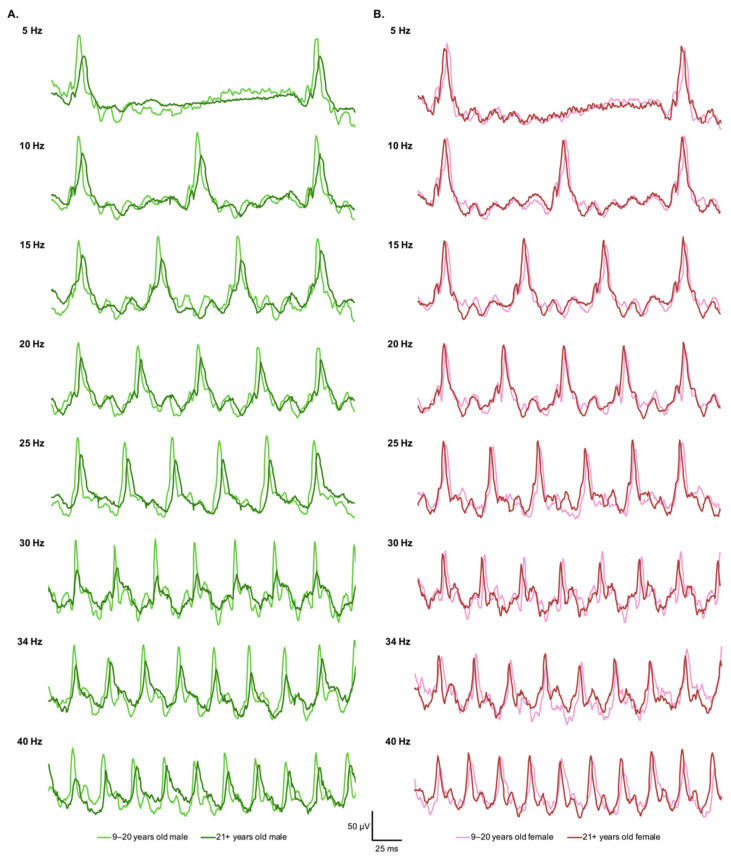
(**A**). Representative flicker ERG traces of a young male subject (light green) and an old male subject (dark green). Note the decrease in the amplitude of the response with aging. (**B**). Representative flicker ERG traces of a young female (pink) and an old female (red) subjects.

Comparison of the cone-driven stimulus-response function between younger and older monkeys disclosed a significant group effect for both the amplitude (*F*(1,33) = 5.495, *p* = 0.025) and the implicit time (*F*(1,33) = 4.435, *p* = 0.043) of the flicker waveform. Specifically, the mean amplitude of the response was reduced (Figure 2A) and the mean implicit time was augmented (Figure 2B) in older monkeys, irrespective of the stimulus frequency tested. Inclusion of sex in a subsequent analysis revealed a significant interaction effect of sex and age group for the amplitude parameter (*F*(1,31) = 4.584, *p* = 0.040). Post hoc pairwise comparisons indicated that the significant age-related decrease in the amplitude of the flicker response was limited to male monkeys (*p* = 0.018) (Figure 3A), with no analogous change observable in female monkeys (*p* = 1.000) (Figure 3B), across all the different repeated flash stimuli tested. Four representative ERG traces are provided to illustrate the variation of the eight light-adapted flicker responses with aging by sex (Figure 4A,B).

## 4. Discussion

This study aimed to unveil the effects of aging on the electrophysiological activity of the retina in a large cohort of vervet monkeys, while investigating for potential sex differences. The cone–bipolar system was isolated by using light flashes of constant intensity flickering at different frequencies, under steady photopic conditions.

Sexually dimorphic responses were identified, suggesting that the progression of events that lead to retinal aging might differ between the sexes. Old female monkeys displayed only a significantly prolonged electrical response, while old male monkeys exhibited alterations in both the temporal and voltage characteristics of the flicker response. Mechanistically, this difference reflects fairly distinct stages of cellular events. While a conduction deficit represents merely an early cell dysfunction, a fall in the amplitude of the electrical response rather indicates a broader retinal impairment, possibly associated with cell death [19,20]. Therefore, based on our results, we can postulate that retinal function is better preserved in aged females.

Circulating sex hormones influence a vast array of body functions. While systemic changes induced by sex hormones (e.g., blood pressure [21]) have the potential to alter the ERG response, a more direct effect is expected in the case of estrogens (reviewed in [22]). In addition to the estrogens reaching the retina through the blood flow, estrogens are locally produced in the retina [23] and estrogen receptors are also expressed therein [24,25,26]. Consequently, the retina is presumably able to sense and to be regulated by the female hormonal environment.

From rats to humans, studies have established correlations between the estrus cycle and retinal functioning, highlighting the contribution of estrogens to the normal retinal physiology [12,14]. As aging becomes the focus, multiple lines of research corroborate the neuroprotective effects of estrogen in the retina [27,28,29,30], as had been earlier proposed for the brain. Generally, the drop in estrogens occurring at menopause is followed by an increased incidence of ocular pathologies, such as age-related macular degeneration, but the incidence and severity are lower among women undertaking estrogen replacement therapy [31,32,33,34]. Furthermore, Chaychi et al. (2015) showed larger ERG responses recorded on premenopausal rats compared to menopausal ones [14]. If one questions why such differences between younger and older vervet females were not replicated by our study, the answer might lie beneath the reproductive characteristics of the vervet monkey. Strikingly, in vervets, reproductive senescence is attained at fairly late stages of life (up to above 23 years of age, where the maximum lifespan of colony-born individuals does not exceed 30 years old) [35]. Hence, considering the age distribution of our older females (21–28 years old, M = 22.6), in most cases it is expectable that they have not reached menopause yet or that they have not been under a prolonged estrogen-deprived state. Accordingly, and for all the reasons discussed above, it is conceivable that structural and functional neurodegenerative changes are delayed and less manifest in the female vervet monkey.

Our study is unique in that it is one of the few studies that looked into such differences in non-human primates, while trying to address the main limitations of the existing literature. In 1980, El-Mofty et al. hinted at a slight and gradual decline in the 35 Hz photopic flicker response with age in a large cohort of semi-natural free-breeding rhesus monkeys [36], though the authors did not quantify this decay nor made any mention of potential sex differences. In 2004, Kim et al. pointed out sex differences in two cohorts of cynomolgus and rhesus monkeys in a study using the multifocal ERG [37]. Perhaps more interesting is the fact that the way the two sexes diverged was opposite between the two species. Unfortunately, the age of the animals employed in this study is not specified. More recently, a small study on 11 cynomolgus monkeys noticed a significant increase in the latency of both the a and b-waves of the photopic ERG with increasing age [38]. However, all monkeys were male and young (4–7 years old).

This study tried to control for other factors that could influence the ERG response, namely, intraocular pressure. However, other elements that could be responsible for altered ERG responses that are usually controlled for in humans were dismissed. This is due to the difficulty to perform an extensive ophthalmological examination in such a large cohort of monkeys in a natural environment where the equipment is inaccessible. For example, refractive errors and optical factors that reduce the effective amount of light eliciting the activity of the retina could be responsible for a diminished recruitment of retinal photoreceptors and, therefore, for a lower amplitude of the ERG waves. In other words, non-neural pre-retinal factors that commonly accompany the aging process, such as losses of mydriatic ability and of lens transparency, could endorse an apparent reduction in retinal response. Kergoat et al. (2001) observed similar attenuation and delay of the flash ERG waves in individuals aged above 75 years old [9]. Relevantly to our study, the authors conducted a subanalysis of the impact of pupillary diameter and media opacity in the measured outcomes and the groups did not differ in any photopically recorded parameter. Congruently, cataracts mostly seem to affect the absorption of short-wavelength light that stimulates rod activity [39]. Moreover, in our study, the average interocular amplitude difference was of 12%, which agrees with the existing literature in visually normal individuals [40] and further supports the inexistence of major ocular pathologies. Accordingly, the findings described here are assumed to exceed the contributions of the optical changes commonly found in the elderly population and to be primarily related to altered neural functioning with aging.

The functional neural changes portrayed here are mirrored by anatomical evidence showing cone photoreceptor cell loss [1,13,41], lack of light-absorbing photopigment present in photoreceptors [42], but also disorganization of the outer layers of the retina [43], seen in both animal models and humans. In addition, while our study focused exclusively on the cone–bipolar axis, comparable age-related changes were reported in the literature for rod-mediated responses [4,5,6,7,8,9,10,13,14]. In fact, some studies have argued for an increased vulnerability of the latter photoreceptor type [44,45], in view of the presence of scotopic a-wave weakening in the absence of alterations of its photopic, i.e., cone-originated, counterpart and even pointed out a reduction in the amplitude of the photopic postreceptoral b-wave not paralleled by changes in the photoreceptor compartment [9,46]. Another study by Freund et al. (2011) observed opposite voltage variations of the dark- and light-adapted a-waves with aging [47]. While the rod-driven response diminished with age like in most studies, the cone-driven response was actually found to be augmented at high stimulus intensities. These discrepancies are most likely due to the different ERG protocols and recording conditions applied, as well as the tested species. Hence, more comprehensive anatomo-functional studies will be needed in the future in order to challenge or to extend these observations to the vervet monkey.

Aside from the intrinsic dysfunction of the photoreceptors and bipolar cells, another possible explanation revolves around the defective function of other structures that play important supportive roles to keep retinal homeostasis, such as the choroid and the retinal pigment epithelium. Changes in vascular supply and neurovascular coupling may underlie some of the alterations in the metabolism and function of retinal cells linked with aging. The degeneration of the retinal pigment epithelium (RPE) with aging entails the progressive inability of this structure to appropriately counteract the photo-oxidative damage of the outer segment of photoreceptors exposed to light and can result in compromised phototransduction. Since both these tissues are also targeted by the normal age-related degenerative processes (reviewed in [48] and [49]), they could contribute indirectly to the deteriorated retinal response identified in older individuals. Furthermore, estrogen receptors are equally present in these structures [24,26,50], which could account for the sex differences.

Altogether, the photoreceptor layer, the RPE and the choroid create a propitious highly oxidative microenvironment, as these tissues have a very elevated metabolic activity, and are continuously exposed to light and to high oxygen concentrations. At the subcellular level, the repetitive oxidative stress results in mitochondrial DNA damage, a molecular hallmark of retina degeneration [51]. With time, the gradual deterioration of the DNA repair and antioxidant systems [52,53] and the development of a para-inflammatory state [54] are thought to further exacerbate mitochondrial dysfunction, ultimately leading to the cell dysfunction seen with aging. Again, estrogens have been found to be protective against the occurrence of this type of injury [28,30].

The research context enabled us to appraise the retinal responsiveness outside the standardized 30 Hz recording protocol defined by the International Society for the Clinical Electrophysiology of Vision (ISCEV), to disclose potential differences that could escape standard testing. Our results, however, were consistent along all the frequencies tested, which suggests the standard 30 Hz flicker clinical measure could be used to discriminate age-related changes.

In summary, our results indicate that age and sex are biological variables that interplay in complex ways to modify the visually elicited electrophysiological phenomena, and thereby emphasize the need to define of age and sex-corrected normative ranges to optimally interpret ERG data, in the research and clinical contexts.

## Data Availability

The data presented in this study are available on request from C.M.-F. (catarina.fernandes@umontreal.ca) and J.B. (joseph.bouskila@umontreal.ca).
